# Cell wall glycans and soluble factors determine the interactions between the hyphae of *Candida albicans* and *Pseudomonas aeruginosa*

**DOI:** 10.1111/j.1574-6968.2008.01301.x

**Published:** 2008-08-01

**Authors:** Alexandra Brand, Julia D Barnes, Kevin S Mackenzie, Frank C Odds, Neil AR Gow

**Affiliations:** Aberdeen Fungal Group, School of Medical Sciences, Institute of Medical Sciences, University of Aberdeen Aberdeen, UK

**Keywords:** *Candida*, cell wall, glycosylation, hypha, *Pseudomonas aeruginosa*

## Abstract

The fungus, *Candida albicans*, and the bacterium, *Pseudomonas aeruginosa*, are opportunistic human pathogens that have been coisolated from diverse body sites. *Pseudomonas aeruginosa* suppresses *C. albicans* proliferation *in vitro* and potentially *in vivo* but it is the *C. albicans* hyphae that are killed while yeast cells are not. We show that hyphal killing involves both contact-mediated and soluble factors. Bacterial culture filtrates contained heat-labile soluble factors that killed *C. albicans* hyphae. In cocultures, localized points of hyphal lysis were observed, suggesting that adhesion and subsequent bacteria-mediated cell wall lysis is involved in the killing of *C. albicans* hyphae. The glycosylation status of the *C. albicans* cell wall affected the rate of contact-dependent killing because mutants with severely truncated *O*-linked, but not *N*-linked, glycans were hypersensitive to *Pseudomonas*-mediated killing. Deletion of *HWP1*, *ALS3* or *HYR1*, which encode major hypha-associated cell wall proteins, had no effect on fungal susceptibility.

## Introduction

*Candida albicans* is a fungus that is part of the normal human commensal microbial communities, but it is also an opportunistic pathogen that can cause life-threatening disseminated infections and superficial mucosal infections such as oral and vaginal thrush. The ability of the fungus to alter its morphology interchangeably between yeast, pseudohyphal and true hyphal growth forms is thought to contribute to pathogenicity (Liu, 2002; Saville *et al.*, 2003; Zheng *et al.*, 2004). Yeast, pseudohyphae and true hyphae have been observed together at infected sites and in biofilms formed by the fungus on the surface of invasive surgical devices (Baillie & Douglas, 1999; Gow, 2002; Netea *et al.*, 2006).

At most body sites, *C. albicans* coexists alongside other microorganisms. The size of the fungal population can be manipulated by suppressing or supporting the growth of commensal enteric bacteria (Payne *et al.*, 2003), suggesting that *C. albicans* proliferation in the gut is controlled by the bacterial microbial communities. *Candida albicans* and the opportunistic bacterial pathogen, *Pseudomonas aeruginosa*, have been coisolated from sputum from patients with cystic fibrosis (CF) (Bakare *et al.*, 2003). *Pseudomonas aeruginosa* and *C. albicans* were also coisolated from patients undergoing surgery and growth of *C. albicans* was observed in sputum samples only after the bacterium had been eradicated (Kerr, 1994). In a study of *C. albicans* and *P. aeruginosa* coisolated from the lungs of CF patients, growth of the fungus was inhibited *in vitro* by the coisolated bacterial strain (Kerr, 1994), suggesting that the bacterium was able to directly suppress the growth of *C. albicans*.

*Pseudomonas aeruginosa* has an extensive repertoire of virulence determinants and is antagonistic to a wide range of microorganisms (Tan *et al.*, 1999). *In vitro* studies of its antifungal activity showed that *P. aeruginosa* inhibited the growth of *Aspergillus fumigatus* and *Candida* spp. (Grillot *et al.*, 1994; Kerr, 1994). The yeast form of *C. albicans* is however resistant to *P. aeruginosa* (Hogan & Kolter, 2002). In a study of direct interactions between a *C. albicans* monomorphic *tup1* mutant that is constitutively hyphal, *P. aeruginosa* was found to form a biofilm on hyphae and to selectively kill them (Hogan & Kolter, 2002). Suppression of fungal growth has been correlated with production of the bacterial phenazine derivatives, pyocynanin and 1-hydroxyphenazine, in culture filtrates (Kerr *et al.*, 1999). Conversely, pyocyanin and the quorum-sensing molecule, 3-oxo-C12 homoserine lactone, can promote growth of *C. albicans* yeast forms *in vitro* (Kerr *et al.*, 1999; Hogan *et al.*, 2004) leading to their escape from *P. aeruginosa* killing. To date it is unclear what mediates colonization and killing of *C. albicans* hyphae by *P. aeruginosa*. In this study, the role of cell-surface glycosyl groups and the presence of associated hypha-specific proteins were evaluated as potential facilitators of contact-mediated killing of *C. albicans* hyphae by *P. aeruginosa*.

## Materials and methods

### Organisms and media

The *C. albicans* strains used in this study are listed in Table 1. *Candida albicans* was maintained on solid YPD [1% (w/v) yeast extract (Oxoid, Unipath, Basingstoke, UK), 2% (w/v) mycological peptone (Oxoid), 2% (w/v) glucose, 2% (w/v) Agar Technical No. 3 (Oxoid)]. *Candida albicans* was grown overnight in SD minimal medium [yeast nitrogen base without amino acids, with ammonium sulfate (Bio101, Carlsbad, California), 2% (w/v) glucose] at 30 °C. Hyphal cells were induced by growth in RPMI-1640. A wild-type *P. aeruginosa* strain, ATCC27853, was used in all experiments and was grown in Luria–Bertani (LB) medium [1% (w/v) tryptone, 1% (w/v) NaCl, 0.5% (w/v) yeast extract] at 37 °C.

**Table 1:** Survival of *Candida albicans* cell surface mutants cultured with *Pseudomonas aeruginosa* in hyphal growth conditions

Strain	Genotype	Description	Mean survival time (days)	References
NGY152	CAI4/CIp10-*URA3*	Control strain	3.7 ± 0.8	Brand *et al.* (2004)
Ca44	*hyr1*Δ	Mutant lacking a hypha-specific cell wall protein	3.7 ± 0.6	Bailey *et al.* (1996)
Ca86	*als3*Δ	Mutant lacking a hypha-specific cell wall protein	3.0 ± 0.0	Hoyer *et al.* (1998)
CAH7-1A1E2	*hwp1*Δ	Mutant lacking a hypha-specific cell wall protein	4.0 ± 0.8	Staab *et al.* (1999)
BCa2-10	*tup1*Δ	Mutant of transcriptional repressor of hypha-specific genes	1.8 ± 0.0*	Braun & Johnson (1997)
NGY337	*mnt1*Δ/*mnt2*Δ	*O*-glycosylation double mutant	2.3 ± 0.5*	Munro *et al.* (2005)
NGY158	*mnt1*Δ	*O*-glycosylation mutant	2.7 ± 0.6*	Munro *et al.* (2005)
NGY145	*mnt2*Δ	*O*-glycosylation mutant	2.7 ± 0.6*	Munro *et al.* (2005)
NGY204	*och1*Δ	*N*-glycosylation mutant	3.7 ± 0.6	Bates *et al.* (2006)
DH15	*mnn4*Δ	Glycosylation mutant lacking phosphomannan in *N*-linked glycan	3.3 ± 0.6	Hobson *et al.* (2004)
NGY355	*pmr1*Δ	Golgi ATPase mutant, partially deficient in *O*- and *N*-glycosylation	3.5 ± 0.5	Bates *et al.* (2005)
NGY146	*mnt3*Δ	*N*-linked glycosyl-transferase mutant	4.0 ± 0.0	Unpublished
NGY313	*mnt4*Δ	*N*-linked glycosyl-transferase mutant	3.0 ± 0.0	Unpublished
NGY147	*mnt5*Δ	*N*-linked glycosyl-transferase mutant	3.7 ± 0.6	Unpublished

*Candida albicans* hyphae were mixed with *P. aeruginosa* ATCC27853 and the viable cell population determined by daily plating. All fungal strains were Ura^+^ (Bain *et al.*, 2001). The mean survival time ± SD (*n*=3) in cocultures was defined as the time point at which the viable concentration of *C. albicans* was reduced to <0.1% of the original population.

* Significant difference from the control (*tup1*Δ*P*=<0.001, *mnt1*Δ*P*=0.027, *mnt2*Δ*P*=0.027, *mnt1*Δ/*mnt2*Δ*P*=<0.001).

### Survival assays

*Candida albicans* cells from overnight cultures were diluted to 1 × 10^6^ cells mL^−1^ in 10 mL RPMI-1640 with 25 mM HEPES and NaHCO_3_ (Sigma-Aldrich, Dorset, UK), supplemented with 0.3 mg mL^−1^l-glutamine, and grown at 37 °C for 3 h to induce hyphal growth. *Pseudomonas aeruginosa* cells from an overnight culture were inoculated into 50 mL 1 : 3 LB : RPMI-1640 (described above) and grown to an OD_650 nm_ of 1.25 at 37 °C. A 25 mL sample was passed through a 0.2 μm PES filter unit (Nalge Nunc International, New York) to generate sterile conditioned medium. Samples consisting of 2.5 mL aliquots of *P. aeruginosa* culture or sterile conditioned medium were added to hyphal cells of *C. albicans* in shake flasks or sterile tubes. The concentration of viable *C. albicans* cells was determined as mean CFUs by plating triplicate 5 μL samples on YPD plates containing tetracycline (60 μg mL^−1^; Progen Industries Ltd, Queensland, Australia), gentamicin (30 μg mL^−1^; Duchefa, Haarlem, the Netherlands) and chloramphenicol (30 μg mL^−1^; Duchefa) to suppress the growth of *P. aeruginosa*. As CFUs decreased, 50 μL aliquots were spread on plates. The limit of detection was *c*. 20 CFU mL^−1^. Plates were incubated for 24–48 h at 30 °C. Results were expressed as the mean time point at which the viable cell concentration decreased to <0.1% of the initial population. Three independent samples were analyzed per strain in each experiment and experiments were carried out on three independent occasions. To compare the survival of cells grown as yeasts, *C. albicans* from an overnight culture was inoculated into YPD, instead of RPMI-1640, and grown at 30 °C for 3 h before addition of *P. aeruginosa*. The anti-*Candida* activity of medium supernatants was determined using 4-day cocultures. Media were filtered with a 0.2-μm PES filter unit, divided into aliquots and *C. albicans* hyphae were inoculated, with or without *P. aeruginosa*, into filtered or autoclaved–filtered media. For controls, *C. albicans* hyphae were inoculated into filtered media from 4-day cultures of the fungus alone. The Dunnett *t*-test in the spss software package (SPSS, Woking, UK) was used for statistical analyses.

### Microscopy

The wells of a sterile Lab-Tek four-well glass slides (Scientific Laboratory Supplies, Nottingham, UK) were coated with fetal bovine serum (Biosera) at 37 °C for 24 h and washed with 0.9 mL phosphate-buffered saline (PBS) (Invitrogen, Paisley, UK). *Candida albicans* yeast cells (1 × 10^5^ cells mL^−1^per well) were incubated at 37 °C for 3 h to induce hypha formation and adhesion. Wells were washed with PBS and 0.9 mL of an exponential-phase *P. aeruginosa* culture was added. Slides were incubated at 37 °C with shaking for up to 3 h. For fluorescence microscopy, 1 μL 10 mM FUN1 LIVE/DEAD stain (Molecular Probes, Leiden, the Netherlands) was added and incubation was continued in the dark for 30 min. Wells were washed with PBS and the well-housing and gasket were removed. Images were captured using an Axioplan 2 microscope (Carl Zeiss Ltd, UK) with a Hamamatsu CCD camera and analyzed with openlab 3.0.9 (Improvision Ltd, Coventry, UK). For light microscopy, images were captured using an Olympus BX50 microscope fitted with an Olympus DP11 camera. For scanning electron microscopy, hyphae and bacteria were cocultured for 48 h. Cultures were filtered through 25-mm polycarbonate filters with 12 μm pores (Costar, High Wycombe, UK) and the cells were fixed with 2.5% glutaraldehyde in 0.1 M phosphate buffer, pH 7.4. Cells were postfixed with 1% osmium tetroxide and dehydrated in 70%, 90%, 95% and 100% ethanol and critical point dried in CO_2_. After sputter-coating with gold, cells were viewed in a JEOL35CF Scanning electron microscope at a voltage of 10 kV.

### Pyocyanin assay

The presence of pyocyanin was measured spectrophotometrically by the method of Essar *et al.* (1990). Briefly, 1 mL of a 6-day culture was extracted in triplicate in 2 mL chloroform and then 1 mL 0.2 M HCl. The OD_520 nm_ was determined and concentration calculated using the pyocyanin molar extinction coefficient. Results were expressed as micrograms of pyocyanin per milliliter of culture (MacDonald, 1967).

## Results

### *Pseudomonas aeruginosa* ATCC27853 kills wild-type *C. albicans* hyphae, but not yeast

It was reported previously that *P. aeruginosa* killed a constitutively hyphal *C. albicans* mutant (*tup1*Δ) within 72 h in M63 medium at 30 °C (Hogan & Kolter, 2002). We found that CAI4/CIp10, a prototrophic control strain in the genetic background used to create most *C. albicans* mutants (Fonzi & Irwin, 1993; Brand *et al.*, 2004), grew as yeasts in these conditions and were not killed by *P. aeruginosa* (data not shown). In order to test wild-type hyphae and a range of *C. albicans* mutants (i.e. non-*tup1*Δ), we grew CAI4/CIp10 in RPMI-1640 at 37 °C. This medium induces the formation of true, nonconstricted hyphae. No reversion to yeast growth was observed over the duration of the experiments, even after 4 days' coculture in the presence of high bacterial densities. *Pseudomonas aeruginosa* grew slowly in RPMI-1640 in the absence of *C. albicans* (data not shown). We compared the viability of the *C. albicans* control strain under hypha-inducing conditions during culture with and without *P. aeruginosa*. Without *P. aeruginosa*, *C. albicans* cultures reached high cell densities and tended to aggregate; hence, the viable cell concentration was likely to be underestimated. On mixing with *P. aeruginosa*, hyphal aggregation was abolished, thereby facilitating CFU counts by plating. However, hyphae consist of multiple cellular compartments; hence, hyphal CFUs are an underestimate of the total number of viable cells present.

**Fig. 1:**
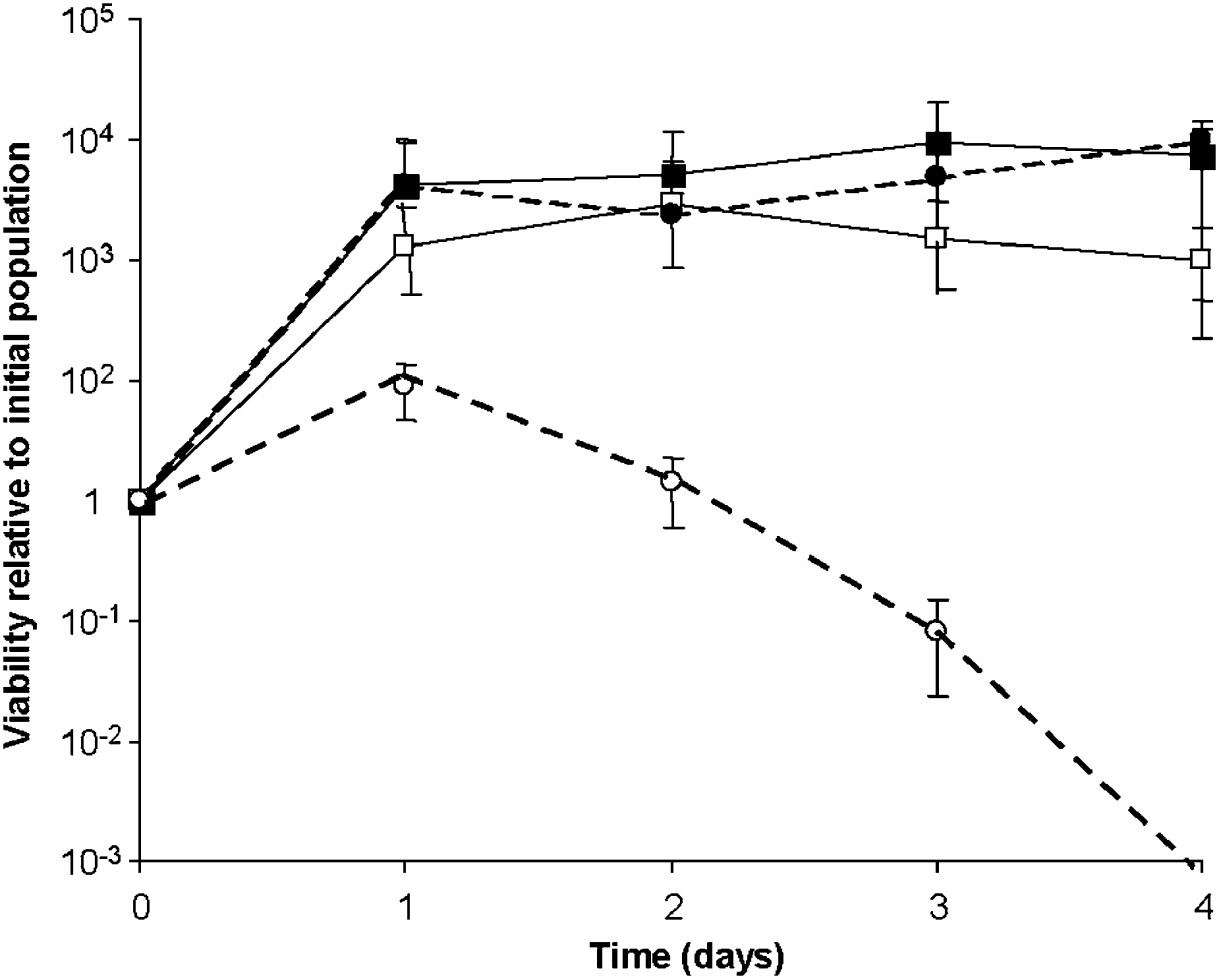
*Pseudomonas aeruginosa* kills *Candida albicans* hyphae, but not yeast. *Candida albicans* yeast cells (1 × 10^6^ cells mL^−1^) were inoculated into 10 mL RPMI-1640 (circles) or YPD (squares) and incubated for 3 h at 37 or 30°C, to produce hyphae or yeast cells, respectively. *Candida albicans* was then cultured for 4 days at 37 or 30°C with (open symbols) or without (closed symbols) the addition of *P. aeruginosa* cells. The fungal viable cell population was determined daily by plating on YPD solid medium containing antibacterial agents. Error bars are SD (*n*=3).

In RPMI-1640, CFUs of the control strain rose and remained in the region of 10^8^ cells mL^−1^ by day 4. When incubated with *P. aeruginosa* ATCC27853, the mean survival time of hyphae was 3.7 days (Fig. 1). The growth rate of *P. aeruginosa* in YPD at 30 °C was twice that in RPMI at 37 °C (data not shown); yet the viable population of *C. albicans* yeast cells, grown with or without the bacterium, increased 60–100-fold within 48 h. By day 4, viable yeast cell concentrations in cocultures declined by a third (Fig. 1). This trend was observed for all *C. albicans* strains growing in the yeast form. The decline of yeast cell viability in YPD cocultures coincided with the appearance of pyocyanin pigment, which reached a mean concentration of 2.2±0.36 μg mL^−1^ (data not shown) by day 4. In contrast, the mean pyocyanin concentration in RPMI-1640 by day 4 was 0.05±0.01 μg mL^−1^. When grown in yeast-inducing conditions, the *tup1*Δ mutant, which is constitutively filamentous, followed the same growth profile as the control strain. Under hypha-inducing conditions, *tup1*Δ was hypersensitive to *P. aeruginosa* (Table 1), consistent with previously reported findings using M63 medium (Hogan & Kolter, 2002).

### Defective *C. albicans O*-glycosylation increases susceptibility to *P. aeruginosa*

Because yeast cells were resistant to *P. aeruginosa*, we investigated whether components of the hypha cell wall influenced susceptibility to the bacterium. Hypha-specific cell wall proteins of *C. albicans* are involved in adhesion and aggregation (Bailey *et al.*, 1996; Hoyer *et al.*, 1998; Staab *et al.*, 1999; Phan *et al.*, 2007) and mannan components of the cell surface have been implicated in adhesion of *C. albicans* to several cell types (Timpel *et al.*, 2000; Dalle *et al.*, 2003; Munro *et al.*, 2005; Bates *et al.*, 2006). Therefore, we tested a range of mutants that lacked hypha-specific cell wall mannoproteins and others that lacked specific glycosyl epitopes. Mutant *C. albicans* strains that lacked the hypha-specific proteins Hyr1p, Hwp1p and Als3p or enzymes involved in *N*-glycosylation (Och1p, Mnn4p, Mnt3p, Mnt4p, and Mnt5p) of surface glycoproteins, were assayed for altered rates of killing by *P. aeruginosa* but none showed increased resistance or sensitivity to the bacterium (Table 1). However, the survival of *mnt1*Δ, *mnt2*Δ and *mnt1*Δ/*mnt2*Δ mutants with truncated *O*-linked mannan was significantly reduced in the presence of *P. aeruginosa* as compared with the control strain, suggesting that *O*-mannan is protective against the *P. aeruginosa* killing activity. Survival of the yeast form of the *O*-glycosylation mutants was the same as the control strain (not shown).

### Medium filtrates have anti-*C. albicans* activity

**Fig. 2:**
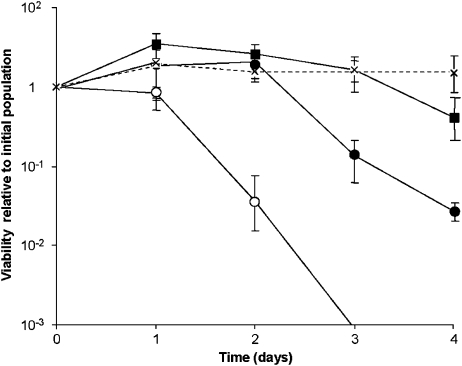
The susceptibility of *Candida albicans* to the presence of *Pseudomonas aeruginosa*, its heat-labile and heat-stable secreted factors. *Candida albicans* hyphae were inoculated into 10 mL cell-free conditioned medium from a 4-day *C. albicans–P. aeruginosa* coculture (Pa–Ca CM). Hyphae were incubated either with (open circles) or without (closed circles) the addition of fresh *P. aeruginosa*. Hyphae were also inoculated into the same filtrate that had been heat treated to denature proteins (Pa–Ca HCM – closed squares) or into filtrate generated from a 4-day *C. albicans*-only culture as a control (Ca-only CM – broken line). Error bars are SD (*n*=3).

We assessed whether the presence of *P. aeruginosa* in the culture medium was required for the killing of *C. albicans* and whether secreted antifungal factors contributed to fungal cell death. *Candida albicans* was inoculated with or without *P. aeruginosa* into filtered conditioned medium, obtained by coculturing the two organisms in RPMI-1640 for 4 days (Pa–Ca CM). *Candida albicans* was also inoculated into the same filtrate that had been heat treated by autoclaving (Pa–Ca HCM) or into a medium in which the fungus alone had been cultured for 4 days (Ca-only CM), as a control. Viable *C. albicans* hyphae were not detectable by day 3 in Pa–Ca CM containing *P. aeruginosa* (Fig. 2) and in the same medium, but without *P. aeruginosa*, *C. albicans* viability declined by 97% by day 4. When inoculated into the same medium that had been heat treated (Pa–Ca HCM), viability fell by 59% by day 4. In contrast, the CFUs for *C. albicans* inoculated into Ca-only conditioned medium increased by 45% over 4 days. The decline in viability in coculture filtrate was therefore likely to be linked to *P. aeruginosa*-specific factors and not to nutrient deprivation. The rate of *C. albicans* cell death was therefore highest in the presence of the bacterium but cell-free filtrates of Pa–Ca CM and Pa–Ca HCM also significantly affected viability (*P*=0.01 and 0.033, respectively).

### *Pseudomonas aeruginosa* adhere to live *C. albicans* hyphae and cause localized cell lysis

**Fig. 3:**
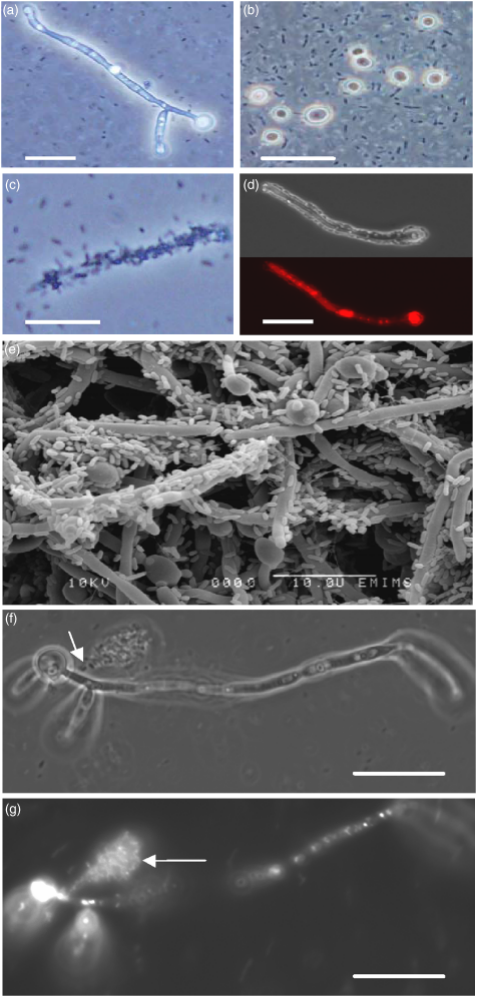
*Candida albicans* hyphae – adhesion, colonization and lysis by *Pseudomonas aeruginosa*. Adhered hyphae were viewed by light microscopy after 3 h coincubation with *P. aeruginosa* in RPMI-1640. Most hyphae and all yeast cells (b) had no observable adherent bacteria (a, b); yet some hyphae were completely colonized by *P. aeruginosa* (c, d). After 48 h, yeast cells and some hyphae were not colonized by *P. aeruginosa* (e). Treatment after colonization with FUN1 LIVE/DEAD red vacuolar stain revealed that hyphae were alive before adhesion by bacteria (d). Cytosol appeared to be released from points on the hyphal surface (arrow, f). The exudate released into the medium (arrow, g) from the point of lysis was confirmed as cytosolic material by FUN1 staining. Scale bar=10 μm.

The interactions between *P. aeruginosa* and *C. albicans* wild-type yeast and hyphae were studied by microscopy. Motile bacteria did not immediately associate with hyphae upon inoculation of *P. aeruginosa* into a hyphal culture of *C. albicans* in RPMI-1640. After 1, 2, 3 and 48 h coincubation in this medium, hyphae were observed with and without attached bacteria (Fig. 3a, c and e). *Pseudomonas aeruginosa* adhered preferentially to specific hyphae at all time points. There appeared to be no preferred attachment site relative to the length of the hypha, the presence or absence of a branch or any other morphological parameter. The vital fluorescent stain FUN1 LIVE/DEAD, applied after colonization, showed that hyphal cell death did not occur before the development of a substantial biofilm on the surface of hyphae (Fig. 3d). Release of intracellular material was observed from sites of bacterial foci on the cell wall (Fig. 3f), suggesting that hyphal lysis occurred as a result of exposure to *P. aeruginosa*. Fluorescent staining of the fungal cytosol confirmed that exudate was released from hyphae into the medium (Fig. 3g). Consistent with previous studies (Hogan & Kolter, 2002), no bacteria were seen adhering to *C. albicans* yeast cells, even after 7 days' coculture (Fig. 3b).

## Discussion

The fungus, *C. albicans*, and the bacterium, *P. aeruginosa*, are frequently coisolated from the sputum of CF patients (Kerr, 1994) and it is therefore possible that the two populations interact directly in the respiratory tract (Hogan & Kolter, 2002). Resistance of the yeast form and susceptibility of the hyphal form of *C. albicans* to killing by *P. aeruginosa* has been reported (Hogan & Kolter, 2002). Induced reversion to the yeast form by *P. aeruginosa* secretion of 3-oxo-C12-homoserine lactone in media that otherwise favored hyphal growth resulted in survival of *C. albicans* (Hogan *et al.*, 2004). Initial studies of this interaction used the transcription factor *tup1* mutant to generate constitutive pseudohyphal growth and thereby avoid the reversion to yeast. We induced hypha formation in a wild-type background in RPMI-1640 medium and were able to cultivate true hyphae that grew without reversion to yeast or pseudohyphal growth for a minimum of 5 days. While the *tup1*Δ mutant was hypersensitive to killing by *P. aeruginosa* under hypha-inducing conditions, it was almost as resistant to killing as the control strain under yeast-inducing conditions. This suggests that yeast-inducing conditions are protective for the constitutively filamentous *tup1*Δ mutant. Under these conditions, *P. aeruginosa* may not produce key antifungal factors, or alternatively, a hypha-specific property required for susceptibility is not induced in the *tup1*Δ mutant. In this case, the susceptibility of *C. albicans* to *P. aeruginosa* is due to a Tup1-independent factor.

Upon inoculation, bacteria did not immediately associate with hyphae and hyphae were not all evenly colonized with bacteria. Instead, bacteria formed foci on selected hyphae. This pattern of selective adhesion by bacterial cells is markedly different from the rapid adhesion of *C. albicans* to host buccal epithelial cells (Watts *et al.*, 1998). Complete colonization of the hyphal surface was observed within 3 h of coincubation. Live/dead staining of hyphae showed that they were viable before colonization. Microscopy revealed points of localized lysis on hyphae where intracellular material escaped into the surrounding medium. Taken together, these observations suggest an active role for *P. aeruginosa* in affecting the integrity of the *C. albicans* cell wall. Cell wall lysis may therefore occur due to the localized accumulation of cytotoxic molecules or degradative enzymes within bacterial foci. The formation of foci of killing by *P. aeruginosa* has also been reported in investigations of adhesion to confluent mammalian cells, suggesting that bacterial aggregation and biofilm formation is important for efficient killing (Apodaca *et al.*, 1995).

Adhesion of *P. aeruginosa* to *C. albicans* is likely to be mediated by the outer, glycoprotein-rich layer of the fungal cell wall. It has been reported that *P. aeruginosa* requires glycoproteins as adhesion sites on host kidney cells (Apodaca *et al.*, 1995). *Candida* glycans are also known ligands for recognition by pattern recognition receptors of the immune system (Netea *et al.*, 2006, 2008). Loss of the hypha-specific proteins Hyr1p, Hwp1p and Als3p or truncation of the wild-type *N*-linked glycan structure did not alter resistance or susceptibility to *P. aeruginosa*. In contrast, mutant hyphae that were deficient in *O*-glycosylation were killed significantly faster than the control strain and the severity of *O*-mannan truncation correlated with increased susceptibility, suggesting that there is a specific role for *O*-glycans in resistance to *P. aeruginosa*. The truncation of *O*-glycans may also result in the exposure of a high-affinity adhesion site for the bacterium or may cause the loss, mislocalization or misfolding of specific surface proteins that are required for fungal cell wall integrity during colonization of *P. aeruginosa*. Therefore, both soluble secreted and insoluble surface factors participate in the selective killing of *C. albicans* hyphae by *P. aeruginosa*.

## Statement

OnlineOpen articles are made available in accordance with the terms of the Creative Commons Deed, Attribution 2.5 (further details available from http://www.creativecommons.org), which allows Open Access dissemination of the article, but does not permit commercial exploitation or the creation of derivative works.
